# Knee osteoarthritis health information on China's TikTok: a cross-sectional analysis of content quality and public health relevance

**DOI:** 10.3389/fdgth.2025.1612749

**Published:** 2026-01-12

**Authors:** Zihan Ding, Jianan Wang, Wangnan Mao, Zheng Yan, Xuchen Zhong, Yintao Du, Lianguo Wu

**Affiliations:** 1 The Second School of Clinical Medicine, Zhejiang Chinese Medical University, Hangzhou, China; 2 The Second Affiliated Hospital of Zhejiang Chinese Medical University (Xinhua Hospital of Zhejiang Province), Hangzhou, China

**Keywords:** health communication, knee osteoarthritis, patient education, social media, TikTok

## Abstract

**Background:**

Knee osteoarthritis (KOA) is a chronic joint disorder that significantly affects the quality of life in the older adult. It is primarily characterized by notable knee pain following activity, which typically alleviates with rest. With the rapid growth of the internet, people increasingly rely on social media to obtain health-related information. Short-form videos, as an emerging format, play an important role in information dissemination. TikTok is currently the world's most downloaded application platform primarily dedicated to short-form video content. Against this backdrop, we observed a substantial number of KOA-related videos on TikTok, the quality and reliability of which have not yet been systematically evaluated.

**Objective:**

To assess the quality and reliability of KOA-related videos available on the domestic TikTok platform.

**Methods:**

A total of 100 KOA-related videos were retrieved and screened from TikTok. Basic metadata were extracted, and video content and format were categorized through coding. The source of each video was also documented. Two independent raters evaluated video quality using the DISCERN instrument, the Journal of the American Medical Association (JAMA) benchmark criteria, and the Global Quality Score (GQS).

**Results:**

Of 100 analyzed videos, 96 were posted by medical staff and 4 by science communicators. Eighty videos were audio-based (41% outpatient daily, 39% general science popularization), with others using graph-text formats. The video content is divided into 7 groups: disease prevention, diagnosis, symptoms, description, life-style and therapy, among which the video related to disease description is the most. The average DISCERN, JAMA and GQS scores of videos were 36.29, 1.24 and 2.45, respectively, and the overall quality was low. Further analysis shows that there are significant differences in video quality between science communicators and medical staff. The number of likes, comments, collections, and shares are strongly positively correlated with each other, and they are weakly positively correlated with the number of upload days and DISCERN scores.

**Conclusion:**

KOA-related content on TikTok demonstrates concerning quality limitations, with significant variation across source types. Given TikTok's expanding influence in health communication, urgent improvements and standardized quality control measures are needed.

## Introduction

Knee osteoarthritis (KOA) is a common chronic joint disorder in orthopedics, characterized primarily by degenerative changes in articular cartilage and secondary osteophyte formation around the joint (including marginal osteophytes and cortical thickening) (1). Its main clinical manifestation is pronounced joint pain after activity (such as level walking or stair climbing), which typically alleviates with rest. The prevalence of KOA increases significantly with age, and women are almost twice as likely to develop KOA as men (2, 3). Moreover, among middle-aged and older adult populations in China, KOA not only imposes a substantial socioeconomic burden (4), but is also often accompanied by psychological issues such as anxiety and depression (5). Therefore, providing patients and the public with accurate, clear health education information on KOA is essential for improving disease awareness, promoting standardized management, and alleviating the associated social burden.

With advances in medicine, the management of KOA has diversified and evolved, ranging from pharmacological treatments and intra-articular injections (6) to surgical interventions such as unicompartmental knee arthroplasty (UKA) and total knee arthroplasty (TKA) (7). In recent years, emerging therapies including mesenchymal stem cells and platelet-rich plasma have also been explored (8–11). When confronted with complex and sometimes contradictory clinical information—for example, evidence that arthroscopic surgery offers limited benefit or is even inferior to conservative management for most KOA patients (12, 13)—patients and their families increasingly require scientific and reliable health information to support decision-making. The internet, particularly social media, has become a primary channel for meeting this need.

With the rapid development of the internet, people increasingly rely on online sources for health-related information. Undeniably, the internet has democratized medicine (14). A wide range of social media platforms (such as YouTube, BiliBili, TikTok, etc.) provide opportunities to expand personal knowledge and enhance individual engagement in health, thereby promoting greater personal initiative (15). Patient health education models have accordingly shifted from the traditional passive, face-to-face physician–patient instruction toward more proactive, internet-based learning (16).

Despite the internet becoming a major channel for information acquisition, relying on it for medical knowledge still presents significant limitations. Some online content creators disseminate misinformation for financial gain or fame. Such false information tends to spread quickly and widely on social media, often due to its novelty and emotional appeal. Moreover, multiple studies indicate that the quality of online health information remains problematic; although some improvements have been observed, reliability is often difficult to guarantee and requires individual discernment (14). Therefore, systematically evaluating the reliability of health information on social media has become an important public health issue.

Short videos are playing an increasingly important role in disseminating information, and their visually engaging and highly shareable nature has made them a growing force in public health education. Among various platforms, TikTok, as a leading global short-video social application, boasts over 1 billion monthly active users (17). Although earlier data indicated that as of September 2021, 47.4% of TikTok users were under the age of 29 (18), recent trends show a diversification in its user demographics (19). This positions TikTok as a potentially significant channel for disseminating health information to a broad audience, including middle-aged and older adult. Given TikTok's platform influence, content dissemination patterns, and the likely attention given to KOA-related topics on the platform, this study selects TikTok as the subject for evaluation. It should be clarified that the present research focuses exclusively on TikTok, aiming to conduct an in-depth analysis of the quality characteristics of KOA-related information on this single platform. Future studies could expand to other platforms such as YouTube or BiliBili for cross-platform comparisons, thereby contributing to a more comprehensive understanding.

Previous studies have begun to examine the quality of TikTok videos related to various orthopedic conditions, such as frozen shoulder, anterior cruciate ligament injuries, and osteoporosis (20–22). However, although observational analysis of KOA-related videos on TikTok has been reported (23), no study has yet systematically evaluated the informational quality of such content using standardized assessment tools. Therefore, this study aims to employ established instruments—including the DISCERN checklist, JAMA benchmark criteria, and Global Quality Scale (GQS)—to systematically assess the quality and reliability of KOA-related videos on the Chinese TikTok platform. The findings intend to reveal current strengths and shortcomings in available content, provide reference for patients, healthcare professionals, and platform administrators, and discuss both the potential and challenges of short-video platforms as channels for disseminating reliable health information.

## Method

### Data retrieval and extraction

On February 16, 2025, we searched the Chinese version of TikTok using the keyword “KOA” and selected the top 124 videos from the search results [previous studies have indicated that analyzing over 100 videos does not significantly affect the outcomes (24), therefore, this scale was adopted for screening]. To minimize potential bias introduced by algorithmic recommendations, the search was conducted using a newly created account.

**Figure 1:**
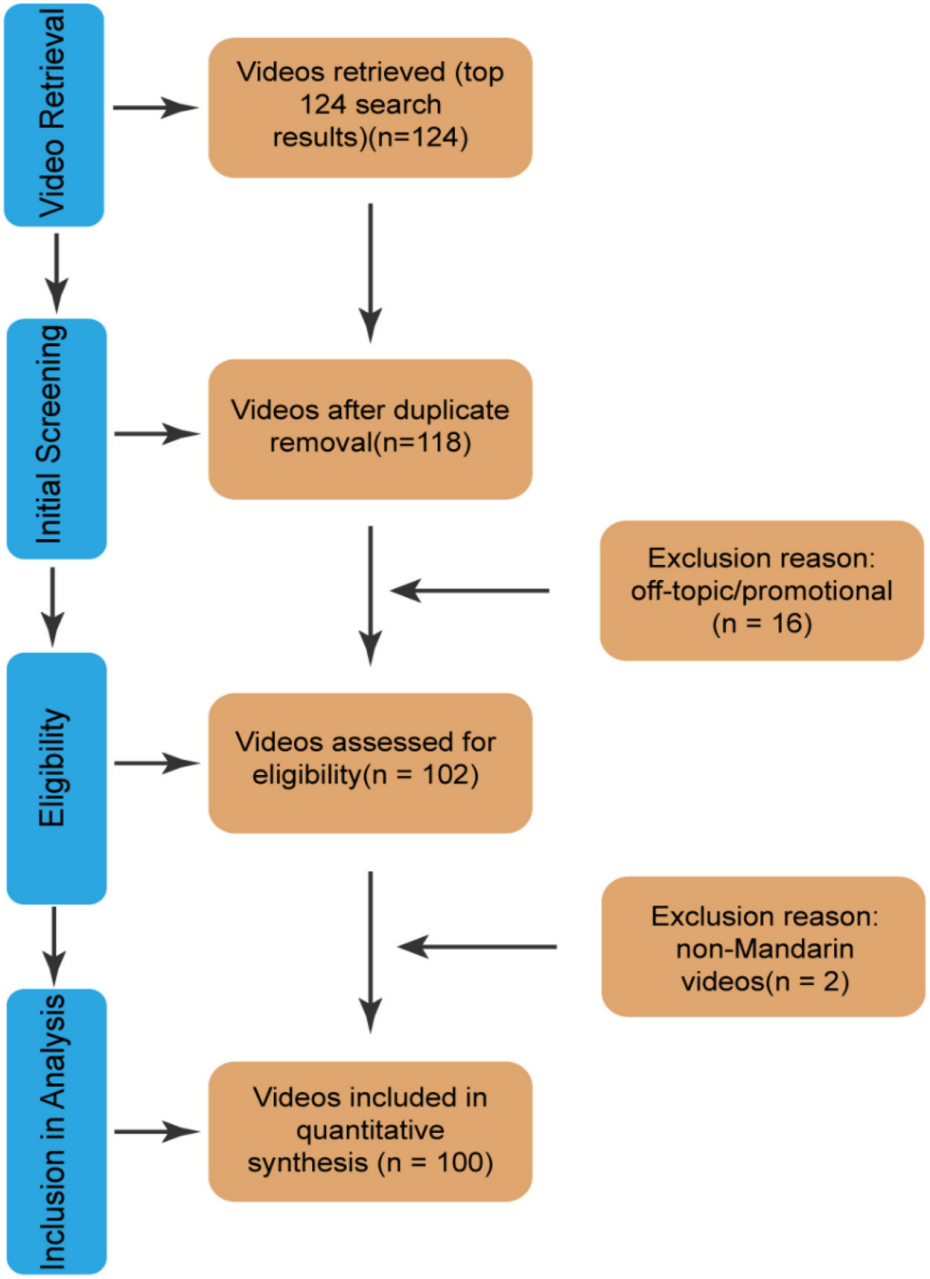
Video screening procedure.

Video screening was performed independently by two reviewers. After viewing all 124 videos, each reviewer excluded ineligible content based on predefined criteria (duplicates, off-topic, advertisement-based, or non-Mandarin Chinese). Through consensus, 100 videos were ultimately included in the analysis. The following metadata were recorded for each video: author, publication date, duration, number of likes, number of favorites, number of comments, and number of shares. All extracted data were documented in an Excel spreadsheet. Figure 1 is the screening flow chart of this study.

### Video classification

This study classified videos along multiple dimensions to systematically analyze their content characteristics and quality variations. Videos were categorized by source based on the publisher's identity into two groups: medical staff (including clinical doctors, nurses, rehabilitation therapists, and other healthcare practitioners) and science communicators (e.g., health science popularization self-media accounts, medical education accounts). Classification primarily relied on account verification information, profile descriptions, and historical content. If such information was unclear, a comprehensive judgment was made by considering the identity of individuals appearing in the video, the professionalism of the narrative, and interactions in the comment section.

Based on presentation format, videos were classified into two types: audio-based (featuring live presenter narration or voice-over, further subdivided into “outpatient daily” and “general science popularization”) and graph-text-based (primarily static images accompanied by textual explanations or subtitles).

Video content was categorized by its core informational theme into six groups, following content classification frameworks used in prior health communication research (22): disease prevention, disease diagnosis, disease symptoms, disease description (a comprehensive introduction to KOA), lifestyle (involving behavioral modifications to delay or manage KOA), and disease therapy (covering introductions to various interventions). Each video was assigned to only one primary content category. Two independent raters performed this categorization; any disagreements were resolved through discussion or by a third reviewer.

To examine trends in video quality over time, videos were divided into four periods based on their publication date: before 2023, 2023, 2024, and 2025. This classification was used solely for the retrospective description of the quantity and quality distribution of content across different periods and was not intended for causal inference or time-series modeling.

## Inter-rater reliability assessment

Prior to formal evaluation, two raters independently conducted a pilot assessment on 20 randomly selected videos. Inter-rater agreement for both classification and scoring was calculated using the Kappa statistic (Kappa > 0.85) (25), indicating good consistency between the raters.

## Quality evaluation methods

### DISCERN instrument

The DISCERN instrument is a reliable and validated tool designed to assess the quality of written consumer health information. It comprises 16 items divided into three sections: the reliability of the information (items 1–8), the quality of the information (items 9–15), and an overall rating (item 16). Each item is scored from 1 to 5 points, yielding a total score ranging from 16 to 80. Based on the total score, quality is categorized as follows: 16–26 (very poor), 27–38 (poor), 39–50 (appropriate), 51–62 (good), and 63–80 (excellent) (26).

### JAMA benchmark criteria

The JAMA benchmark criteria represent a widely used tool for evaluating medical information from online sources. This tool assesses four key dimensions: authorship, attribution, disclosure, and timeliness. Each criterion is assigned 1 point if adequately met, resulting in a total score ranging from 0 to 4. Scores are interpreted as follows: 0–1 indicates insufficient information, 2–3 indicates partially sufficient information, and 4 indicates completely sufficient information (27).

### Global quality score

The Global Quality Score (GQS) is a rating system developed by Bernard et al. to evaluate the overall usefulness of video content for viewers. It assesses the quality of information, media presentation, and user-friendliness. The GQS is scored on a 5-point scale, where 1 represents poor quality with no utility for the target audience, and 5 represents excellent quality with high utility for the target audience (28).

Two independent raters evaluated all included videos using the DISCERN instrument, JAMA benchmark criteria, and GQS. In cases of scoring discrepancies, a third reviewer performed an additional assessment. The final score for each video was determined based on majority consensus or the average of the ratings.

## Statistical analysis

Descriptive statistics were presented as medians, means, interquartile ranges (IQR), and standard deviations (SD). Given the categorical nature of the dataset and non-normal distribution of the data, the Mann–Whitney *U* test was used to assess significant differences between two independent groups, and the Kruskal–Wallis test was applied for comparisons among multiple independent groups. Correlations between parameters were evaluated using the Spearman correlation coefficient. All statistical analyses were conducted using GraphPad Prism (version 10.1.2) and R (version 4.4.2). A *P*-value < 0.05 was considered statistically significant.

## Result

### Video features

**Figure 2:**
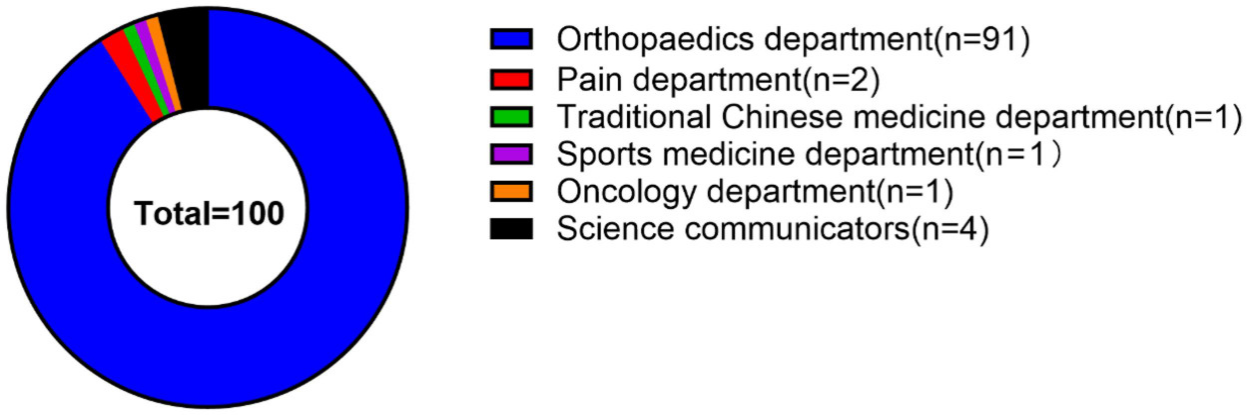
Proportion of different video sources.

Of the analyzed videos, the majority were published by medical staff (96%, 96/100), followed by science communicators (4%, 4/100). Among the medical staff contributors, orthopedic surgeons constituted the largest subgroup (91%, 91/100). The remaining videos were distributed across other specialties, including pain medicine (2%, 2/100), traditional Chinese medicine (1%, 1/100), sports medicine (1%, 1/100), and oncology (1%, 1/100) (Figure 2).

**Figure 3:**
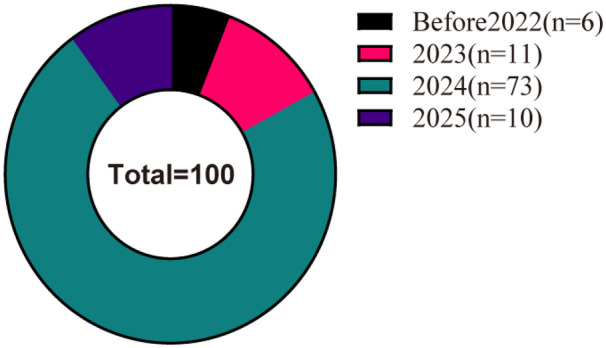
Statistics of videos released in different years.

The distribution of videos by publication year showed that the largest number were released in 2024 (*n* = 73), followed by 2023 (*n* = 11), 2025 (*n* = 10), and the period before 2023 (*n* = 10) (Figure 3).

Regarding video format, outpatient daily videos constituted the largest proportion (41%, 41/100), followed by general science popularization (39%, 39/100) and graph-text presentations (20%, 20/100). In terms of content, disease description was the most frequently discussed topic (37%, 37/100), followed by disease therapy (31%, 31/100), lifestyle (12%, 12/100), disease diagnosis (10%, 10/100), disease prevention (6%, 6/100), and disease symptoms (4%, 4/100).

Video duration ranged from 6 s to 1,535 seconds. The median video length was significantly longer for science communicators (153.5 s) compared to medical staff (56.5 s). The earliest video was published on August 4, 2021 (1,302 days before data collection), and the most recent on February 15, 2025 (one day before data collection). Most videos were published within the past year (median: 196 days). Engagement metrics varied widely: likes ranged from 8 to 253,000, comments from 1 to 6563, collections from 1 to 265,000, and shares from 1 to 95,000 (Tables 1 and 2).

**Table 1:** General characteristics of the videos.

Characteristics	Minimum value	Maximum value	Mean
Video duration (seconds)	6	1,535	107.93
Number of likes	8	2,53,000	11,607.4
Number of comments	1	6,563	420.03
Number of collections	1	2,65,000	5,831.27
Number of shares	1	95,000	5,019.23
Days on TikTok	1	1,302	270.11
DISCERN	20	54	36.29
JAMA	1	2	1.24
GQS	1	5	2.45

JAMA, journal of the American medical association; GQS, global quality score.

**Table 2:** Descriptive statistics of TikTok videos in different categories.

Variables	Video duration (seconds), median (IQR)	Number of likes, median (IQR)	Number of comments, median (IQR)	Number of collections, median (IQR)	Number of shares, median (IQR)	Days on TikTok, median (IQR)
Video source (*n* = 100)						
Medical staff (*n* = 96)	56.5 (38–91)	1,774.5 (333–5,420)	96 (17–257)	654 (127–2,297)	313 (49–1,246)	196 (110–332)
Science communicators (*n* = 4)	153.5 (120–301)	30,500 (7,552 −1,01,500)	889.5 (165 -2,138)	25,386 (4,383 -10,000)	21,779 (2,743 -51,500)	93 (70 -247)
Video format (*n* = 100)						
Outpatient daily(*n* = 41)	56 (43–89)	2,178 (555–6,897)	141 (50–333)	574 (155–2,191)	262 (73–1,222)	198 (120–322)
General science	88 (58–166)	3,240 (608.5–9,361)	162 (47–356)	1,895 (194–4,210.5)	1,018 (237 -2,165)	294 (93–496)
Popularization (*n* = 39)						
Graph-text (*n* = 20)	20 (16–31)	253.5 (120–729)	11.5 (6–37)	149 (57–454)	63.5 (19 -258)	147 (104–207)
Video content (*n* = 100)						
Prevention (*n* = 6)	47.5 (24–53)	3,745 (547–9,145)	96.5 (22–234)	319.5 (434–6,292)	2,040.5 (154–4,212)	262.5 (201–702)
Diagnosis (*n* = 10)	47 (23–101)	590 (152–4,293)	11.5 (8–290)	275 (87–2,135)	134.5 (22–848)	154 (67–230)
Symptoms (*n* = 4)	94.5 (58–139)	1,516 (302–2,914)	76 (28–133)	562 (171–1,465)	610 (151–1,227)	264 (156–421)
Life-style (*n* = 12)	52.5 (38–85)	2,379 (666–5,513)	113.5 (76–315)	1,273.5 (314–2,749)	466.5 (201–1,741)	144 (96–302)
Description (*n* = 37)	88 (53–177)	1,420 (338–3,979)	80 (25–215)	524 (124–1,866)	267 (58–1,067)	173 (92–326)
Therapy(*n* = 31)	49 (34–87)	2,936 (376–12,861)	161 (36–699)	979(152–3,506)	689(70–4,407)	201(112–376)

IQR, interquartile distance, All data are rounded.

### Quality assessment

The overall quality of the evaluated videos was low, with mean scores of 36.29 (DISCERN), 1.24 (JAMA), and 2.45 (GQS). Analysis by video source showed that content from science communicators received higher scores across all three instruments compared to videos from medical staff (Mean JAMA: 1.75 vs. 1.22; Mean GQS: 3.5 vs. 2.41; see Table 3).

**Table 3:** Quality assessment results of different video sources.

Video source(*n* = 100)	Information reliability, mean (SD)	Information quality, mean (SD)	Overall rating, mean (SD)	Total score, mean (SD)	JAMA, mean (SD)	GQS, mean (SD)
Medical staff (*n* = 96)	21.34 (2.93)	11.73 (3.74)	2.80 (1.19)	35.88 (6.20)	1.22 (0.42)	2.41 (0.64)
Science communicators(*n* = 4)	25.25 (0.96)	16.50 (1.91)	4.50 (0.58)	46.25 (2.87)	1.75 (0.50)	3.5(0.58)

**Figure 4:**
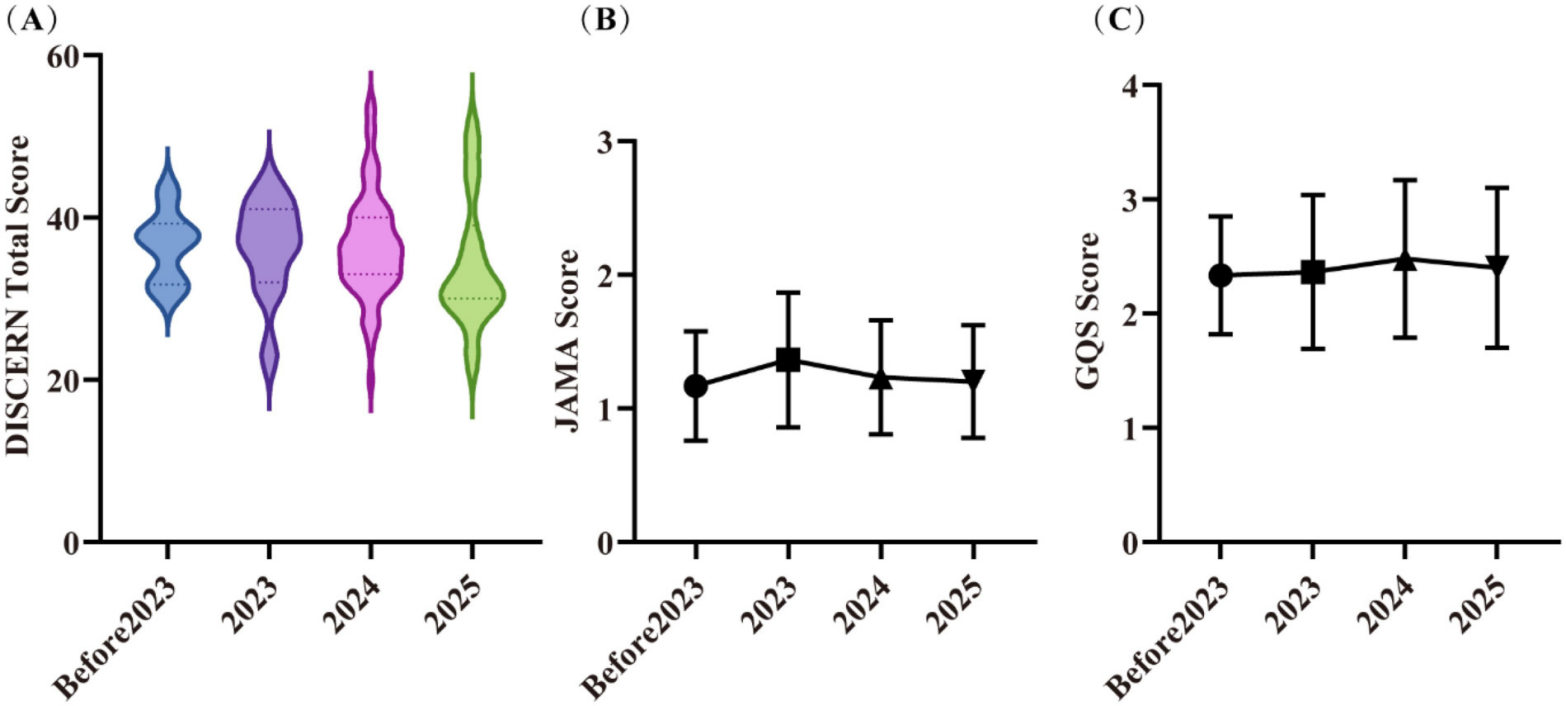
Video quality in different years. **(A)** DISCERN scores; **(B)** JAMA scores; **(C)** GQS scores. Error bars represent standard deviations. Part labels **(A)**, **(B)** and **(C)** correspond to the three subplots illustrating quality scores across years (Before 2023, 2023, 2024, 2025).

According to Figure 4, overall video quality remained consistently poor across the study period, with no statistically significant difference observed between years. Pre-2023: Score distributions were narrow and concentrated in the lower range, indicating uniformly low quality. 2023–2024: The score distribution widened, with an increase in mid-range scores and the emergence of higher-scoring videos, suggesting an initial diversification in quality. 2025: The distribution expanded further, showing a notable increase in the proportion of high-scoring videos. However, this period also exhibited increased polarization, as the quality of low-scoring videos declined compared to previous years. This contributed to the lowest mean DISCERN score (34) being recorded in 2025.

Analysis by video format revealed consistent trends across the DISCERN subscores (Table 4). For the reliability subsection, general science popularization videos achieved the highest mean score (22.95), followed by graph-text-based videos (20.95) and outpatient daily videos (20.39). In the information quality subsection, general science popularization videos again achieved the highest mean score (12.97), ahead of outpatient daily videos (12.24) and graph-text-based videos (9.20). The overall rating subsection followed the same ranking order, with general science popularization videos at 3.59, followed by graph-text-based videos (2.75) and outpatient daily videos (2.24).

**Table 4:** Quality assessment results of different video formats.

Video format (*n* = 100)	Information reliability, mean (SD)	Information quality, mean (SD)	Overall rating, mean (SD)	Total score, mean (SD)	JAMA, mean (SD)	GQS, mean (SD)
Outpatient daily(*n* = 41)	20.39 (2.57)	12.24 (3.37)	2.24 (0.97)	34.88 (5.71)	1.07 (0.26)	2.22 (0.65)
General science popularization(*n* = 39)	22.95 (2.69)	12.97 (4.28)	3.59 (1.04)	39.51 (6.52)	1.46 (0.51)	2.74 (0.68)
Graph-text (*n* = 20)	20.95 (3.25)	9.20 (2.14)	2.75 (1.29)	32.90 (4.90)	1.15 (0.37)	2.35(0.49)

**Figure 5:**
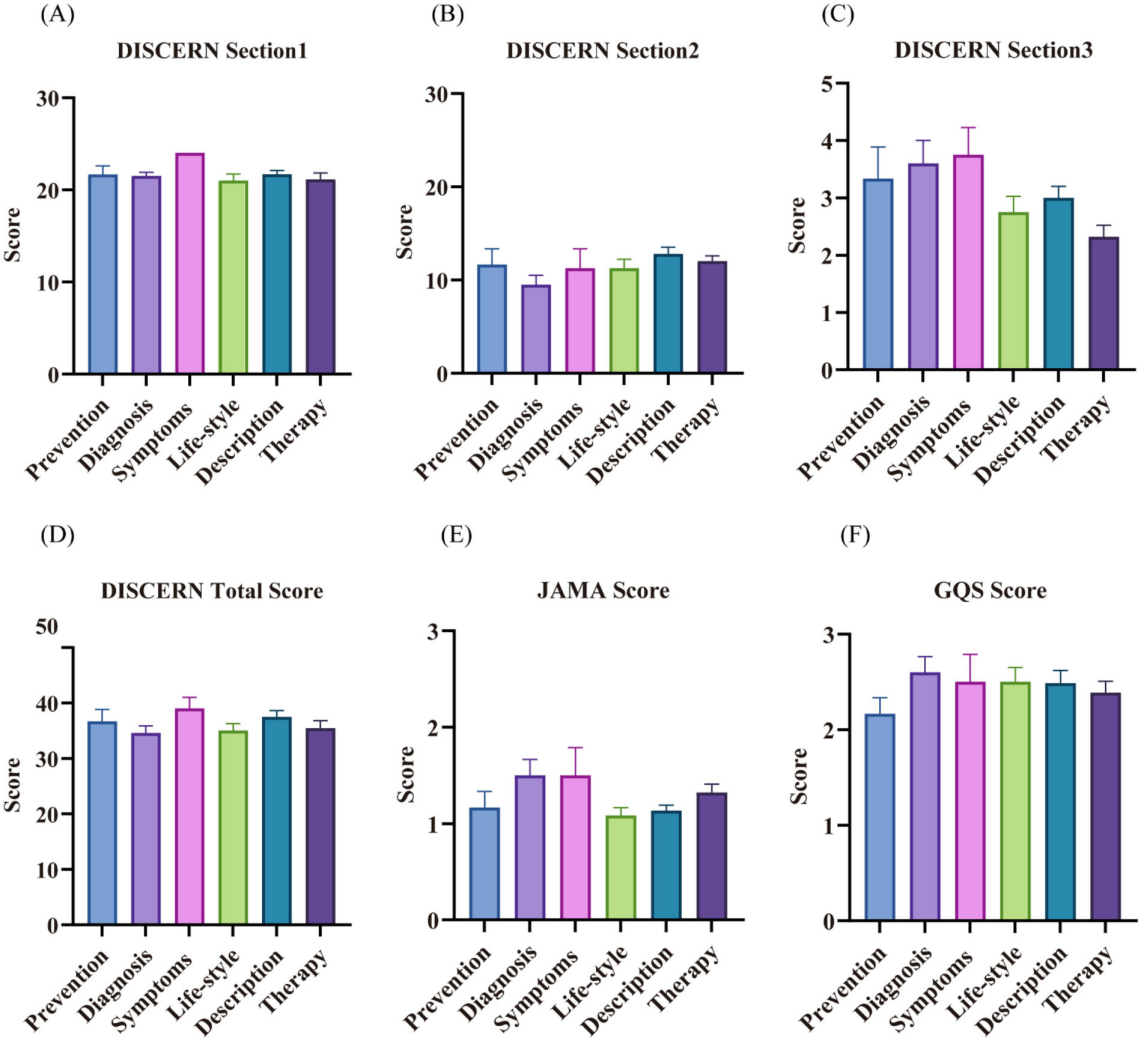
Results of the quality assessment of different video contents. **(A)** DISCERN Section 1 (Information reliability) scores; **(B)** DISCERN Section 2 (Information quality) scores; **(C)** DISCERN Section 3 (Overall rating) scores; **(D)** DISCERN total scores; **(E)** JAMA scores; **(F)** GQS scores. Error bars represent standard deviations. Part labels **(A–F)** respectively correspond to the subplots analyzing quality performance across six video content categories (Prevention, Diagnosis, Symptoms, Life-style, Description, Therapy).

Analysis by video content category showed numerical variations in DISCERN total scores (Figure 5). The highest total DISCERN score was observed for videos on disease symptoms (mean = 39.00), followed by disease overview (37.50), disease prevention (36.67), disease therapy (35.48), lifestyle (35.00), and disease diagnosis (34.60). For the reliability subsection, videos focusing on disease symptoms scored the highest (mean = 24.00), while scores for other categories were clustered around 21.00 points. In the information quality subsection, disease overview videos had the highest score (mean = 12.81), and disease diagnosis videos the lowest (9.50). For the overall rating, disease symptom videos scored the highest (3.75), while disease therapy videos scored the lowest (2.32). Notably, regardless of content category, both JAMA (mean = 1.24) and GQS (mean = 2.45) scores remained consistently low across all videos.

**Figure 6:**
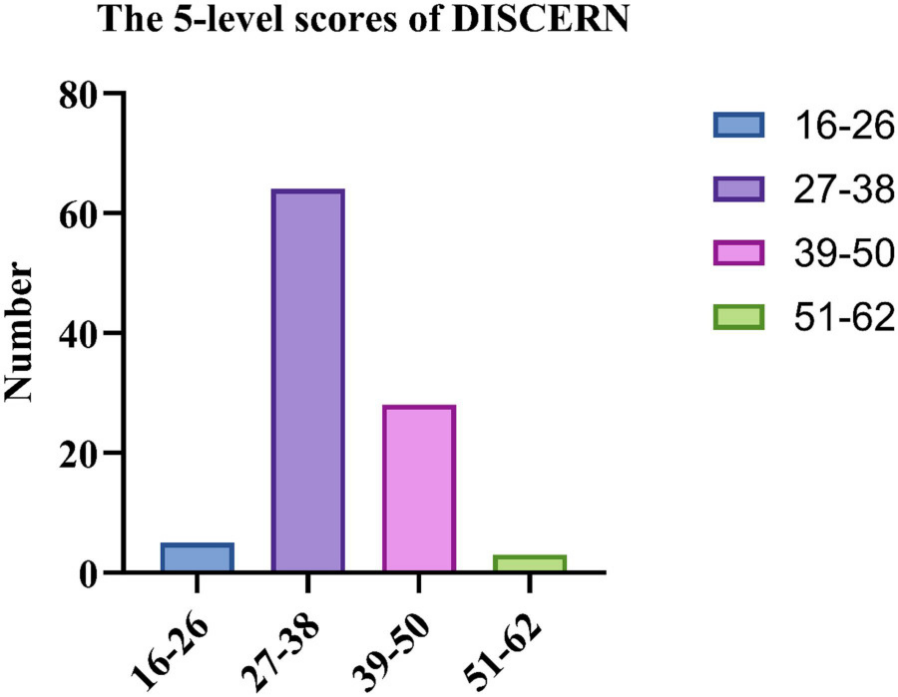
The 5-level scores of DISCERN.

Based on DISCERN total score categories, the majority of videos were of poor quality: 1 video (1.0%) scored 16–26 (very poor), 64 videos (64.0%) scored 27–38 (poor), 28 videos (28.0%) scored 39–50 (appropriate), and only 3 videos (3.0%) scored 51–62 (good) (Figure 6). This indicates that most KOA-related videos on the platform were of low quality, with only a small proportion rated as good.

### Difference analysis

Using R (version 4.4.2), the collected video samples were analyzed with video source, video format, and video content as independent variables, and DISCERN, JAMA, and GQS scores as dependent variables. The results showed no statistically significant differences among the various video content groups. However, significant differences were observed between medical staff and science communicators in DISCERN (*P* = 0.003), JAMA (*P* = 0.015), and GQS scores (*P* = 0.002), with videos released by science communicators demonstrating significantly higher quality than those by medical staff (Table 5).

**Table 5:** Results of the difference analysis of different video sources.

Video source (*n* = 100)	Medical staff (*n* = 96) Score,mean(SD)	Science communicators (*n* = 4) Score,mean(SD)	*P*
DISCERN	35.88 (6.20)	46.25 (2.87)	**0** **.** **003**
JAMA	1.22 (0.42)	1.75 (0.50)	**0** **.** **015**
GQS	2.41 (0.64)	3.5 (0.58)	**0** **.** **002**

Bold *P* values indicate statistically significant differences between groups (*P* < 0.05).

Significant differences were observed between outpatient daily and general science popularization videos in DISCERN (*P* = 0.001), JAMA (*P* < 0.001), and GQS scores (*P* < 0.001). A significant difference was also found in DISCERN scores between general science popularization and graph-text formats (*P* = 0.0002). In contrast, no significant difference was detected between outpatient daily and graph-text videos. Overall, the quality of general science popularization videos was relatively higher (Table 6).

**Table 6:** Results of the difference analysis of different video formats.

Score, mean(SD)	Outpatient daily (*n* = 41)	General science popularization (*n* = 39)	Graph-text (*n* = 20)	P		
				Outpatient daily/General science popularization	Outpatient daily/Graph-text	General science popularization/ Graph-text
DISCERN	34.88 (5.71)	39.51 (6.52)	32.90 (4.90)	**0** **.** **001**	0.939	**0** **.** **0002**
JAMA	1.07 (0.26)	1.46 (0.51)	1.15 (0.37)	**<0** **.** **001**	1	0.05
GQS	2.22 (0.65)	2.74 (0.68)	2.35 (0.49)	**<0** **.** **001**	0.41	0.09

Bold *P* values indicate statistically significant differences between groups (*P* < 0.05).

### Correlation analysis

Spearman correlation analysis was performed using R (version 4.4.2) to examine the relationships among video engagement metrics (likes, comments, collections, shares), time since publication (days on TikTok), and quality assessment scores (DISCERN, JAMA, GQS). The results indicated strong positive correlations between likes, comments, collections, and shares. Weak positive correlations were observed between days on TikTok and each of these engagement metrics, as well as between DISCERN scores and the engagement metrics. Additionally, DISCERN score showed weak positive correlations with both JAMA and GQS scores (Table 7).

**Table 7:** Correlation analysis of video variables.

Variable and analysis	Likes	Comments	Collections	Shares	Day on TikTok	DISCERN	JAMA	GQS
Likes								
Correlation coefficient	1	—	—	—	—	—	—	—
*P* value	—	—	—	—	—	—	—	—
Comments								
Correlation coefficient	0.942	1	—	—	—	—	—	—
*P* value	**<0** **.** **001**	—	—	—	—	—	—	—
Collections								
Correlation coefficient	0.952	0.856	1	—	—	—	—	—
*P* value	**<0** **.** **001**	**<0** **.** **001**	—	—	—	—	—	—
Shares								
Correlation coefficient	0.945	0.901	0.949	1	—	—	—	—
*P* value	**<0** **.** **001**	**<0** **.** **001**	**<0** **.** **001**	—	—	—	—	—
Day on TikTok								
Correlation coefficient	0.329	0.33	0.275	0.377	1	—	—	—
*P* value	**<0** **.** **001**	**<0** **.** **001**	**0** **.** **005**	**<0** **.** **001**	—	—	—	—
DISCERN								
Correlation coefficient	0.272	0.233	0.336	0.348	—	1	—	—
*P* value	**0** **.** **006**	**0** **.** **001**	**<0** **.** **001**	**<0** **.** **001**	—	—	—	—
JAMA								
Correlation coefficient	—	—	0.217	—	—	0.385	1	—
*P* value	—	—	**0** **.** **029**	—	—	**<0** **.** **001**	—	—
GQS								
Correlation coefficient	0.229	—	0.296	0.303	—	0.506	0.405	1
*P* Value	**0** **.** **021**	**—**	**0** **.** **002**	**0** **.** **002**	**—**	**<0** **.** **001**	**<0** **.** **001**	—

Bold *P* values indicate statistically significant differences between groups (*P* < 0.05).correlation coefficient: the larger the value, the stronger the correlation.

## Discuss

### Main findings

This study analyzed the top 100 KOA-related videos on TikTok. The majority of videos were published within the past year (median: 196 days), with the highest volume observed in 2024 (*n* = 73), indicating a growing presence of KOA-related content on the platform. Aggregated user engagement metrics reflected substantial reach, totaling 116,074 likes, 58,312 collections, 50,192 shares, and 42,003 comments. These findings support the potential of TikTok as a channel for disseminating health education.

Medical staff constituted the primary publishing group (96%), with orthopedic surgeons representing the largest subgroup (91%). This represents a notable increase compared to the proportion reported by Hong et al. (67% medical staff) (23)., suggesting that Chinese healthcare professionals are actively engaging in KOA knowledge dissemination via TikTok, which may contribute to the gradual enhancement of the platform's credibility in health communication.

The mean DISCERN (36.29), JAMA (1.24), and GQS (2.45) scores indicate that the overall quality of the videos is relatively low. While these videos may serve an educational or informational purpose for non-medical audiences (such as patients and their families), their reliability and informational quality remain substantially inadequate, requiring viewers to exercise careful discernment. These findings are consistent with those reported in previous studies (20, 22, 29).

From a temporal perspective, no significant change was observed in overall video quality over time, as indicated by the lack of statistical difference between years. However, isolated cases of higher-quality videos began to emerge from 2023 onward. By 2025, the distribution of video quality became markedly polarized: while the number of high-scoring videos increased, the quality of low-scoring videos declined compared to earlier years. This trend may reflect a growing number of creators who prioritize content quality. Nevertheless, the visibility of higher-quality content appears limited, potentially due to competition with or algorithmic dilution by lower-quality videos. This dynamic could dampen the motivation of quality-conscious creators. Consequently, our findings suggest that health-related content on TikTok may benefit from the implementation of quality oversight mechanisms.

From the perspective of video content, videos focused on disease symptoms achieved the highest total DISCERN score (mean = 39), followed by those on disease description (37.5) and disease prevention (36.67), while videos related to disease diagnosis scored the lowest (34.6). This indicates that on TikTok (Douyin), videos introducing disease symptoms and general disease overviews exhibit relatively higher information completeness and reliability, whereas content related to diagnosis is generally of lower quality. This disparity may lead to misunderstandings during patients' self-assessment of their condition, potentially exacerbating their anxiety. Therefore, it is recommended that content creators—particularly physicians—prioritize symptom description, disease mechanisms, and preventive measures when producing KOA-related videos. Diagnostic criteria and evidence-based treatment options should be presented in a clear and accessible manner, avoiding oversimplification, overgeneralization, or misleading claims.

Furthermore, although therapy-related videos were numerous (31%), their overall DISCERN score was moderate (35.48), and they received the lowest rating in the “overall evaluation” subsection (2.32). This suggests significant room for improvement in the comprehensiveness and practicality of such content. Healthcare professionals and science communicators are advised to clearly distinguish between evidence-based treatments and experimental therapies when discussing management options and to emphasize the importance of making treatment decisions under professional medical guidance.

The difference analysis revealed significant disparities between videos posted by science communicators and medical staff (DISCERN: *P* = 0.003; JAMA: *P* = 0.015; GQS: *P* = 0.002), with content from science communicators demonstrating notably higher quality. Additionally, videos by science communicators were longer in duration (median: 153.5 s vs. 56.5 s) and achieved higher user engagement in terms of likes, comments, collections, and shares. These findings suggest that medical staff may not have devoted adequate attention to video quality, contributing to the generally low quality of KOA-related content on the platform. Notably, the results contrast with certain prior studies (30, 31), which reported higher video quality from physicians in other disease areas. In the present study, although medical professionals constituted the majority of publishers (96%), their video quality was not superior to that of science communicators. Similar conclusions have been reported in evaluations of health information videos on YouTube (32), supporting the broader relevance of this observation.

We propose that the observed differences may arise from the following factors:

Communication style: Science communicators often prioritize audience comprehension and information dissemination, employing accessible language, structured delivery, and visual aids to improve information retention. In contrast, medical staff may emphasize clinical accuracy without fully adapting to the communicative norms of short-video platforms.
Content production expertise: Science communicators typically possess stronger media production and narrative skills, allowing them to translate complex medical knowledge into coherent and engaging audiovisual content, thereby enhancing viewer experience and educational impact.
Audience targeting: Science communicators usually design content for non-specialist audiences, aligning with the informational needs and cognitive levels of patients and their families. Medical staff, however, may be inclined to share clinical vignettes or brief reminders rather than producing structured, patient-oriented educational material.

These insights suggest that medical professionals engaging in health communication via social media could benefit from adopting strategies used by science communicators. While maintaining medical rigor, efforts should be made to improve the accessibility, organization, and audience appeal of the content.

A study by Onder et al. on osteoporosis-related videos on YouTube reported that nearly half were of high quality, approximately one-third were of moderate quality, and only a small proportion were of lower quality; video durations ranged from 2.54 to 16 min (33). In contrast, research by Li et al. on osteoporosis-related content on TikTok found that nearly half of the videos were of low quality, with only a minority rated as high quality, and video lengths varied from 9 to 379 s (22). This discrepancy suggests that the shorter video format typical of TikTok may limit the effective delivery of comprehensive disease-related information, thereby compromising content quality (34). Consequently, we encourage healthcare video creators—particularly physicians, who represent the largest and most authoritative group of publishers—to consider extending video duration where appropriate, condensing key messages, and striving to enhance overall content quality.

Some prior studies excluded silent (graph-text) videos (35) or categorized videos only into two broad groups: audio and silent (36).In our sample, however, a notable proportion of audio videos presented content in the format of outpatient consultations (41/100, 41%). Therefore, we classified this subtype separately to examine its specific influence on video quality.

The relatively high proportion of outpatient consultation-style videos may be attributed to their perceived relatability for viewers and the lower production burden for medical staff, as they often can be recorded during fragmented clinical time. However, the quality of these videos, as reflected in the total DISCERN score (34.88), was lower than that of general science popularization videos (39.51). Furthermore, graph-text videos scored the lowest among the three formats in terms of both engagement metrics (duration, likes, comments, shares, collections) and quality scores (DISCERN, JAMA, GQS). These findings align with prior studies (20, 21, 36), suggesting that graph-text content generally exhibits the poorest quality and reach for most disease topics on TikTok. Therefore, we recommend that medical staff consider reducing the publication of both outpatient consultation and graph-text videos, while increasing the proportion of conventional science popularization content to enhance the overall quality of KOA-related information on the platform. Concurrently, healthcare professionals should engage with social media more deliberately. As a group perceived by the public as authoritative, they must recognize their responsibility to provide accurate and effective information from a professional standpoint. When necessary, medical professionals should actively develop competencies in digital professionalism to mitigate potential adverse consequences associated with social media use, such as diluted accountability, blurred professional boundaries, breaches of confidentiality, unprofessional behavior, and related legal or disciplinary risks (37).

Prior studies have indicated that metrics such as likes, comments, shares, and collections reflect the popularity and reach of a video (38, 39). In our Spearman correlation analysis, strong positive correlations were observed among these engagement indicators, suggesting that higher values correspond to greater video popularity. Furthermore, the number of days since upload also showed a positive correlation with popularity, implying that visibility tends to increase over time. Additionally, the DISCERN score exhibited a positive correlation with popularity, indicating that videos of higher quality tend to achieve broader reach. The JAMA score was positively correlated with the number of collections, and the GQS score was positively correlated with likes, collections, and shares. These results align with the findings reported by D'Ambrosi et al. (21) but differ from those of Sun et al. (40). This pattern may reflect a gradual improvement in the public's ability to discern video quality over time, potentially due to rising educational levels and media literacy. Consequently, the role of social media in disseminating evidence-based health information is likely to expand in influence.

This study highlights the dual role of social media in health information dissemination within an aging society. As China's population ages at an accelerating pace, platforms such as TikTok serve as important channels for the dissemination of chronic disease health knowledge, offering significant public health value, especially for education on high-risk conditions prevalent among older adult, such as KOA. However, the study identified two major issues in the current content landscape. First, a substantial proportion of TikTok health content is of low quality. Given its role as an important source of health information for middle-aged and older adult, such content may lead to misunderstandings regarding disease management strategies, delays in appropriate treatment, or reliance on ineffective interventions. Second, although medical professionals constitute the majority of content producers (96%), their effectiveness in communication and the rigor of information shared on social media require urgent improvement. Therefore, to harness the positive potential of social media in public health, a coordinated multi-stakeholder approach is needed. This should include enhancing oversight of platform algorithms to prioritize the promotion of authoritative, high-quality content, alongside strengthening the digital media literacy of healthcare professionals to facilitate the accurate and accessible translation of evidence-based medical knowledge for public communication.

## Limitations and future directions

This study has several limitations. First, our analysis was confined to KOA-related videos on the Chinese version of TikTok and did not include content from other countries, which may introduce selection bias. Second, the cross-sectional design captures video quality only at a specific point in time and cannot infer long-term trends in the quality of KOA-related content. Furthermore, we focused solely on TikTok and did not examine platforms such as YouTube or BiliBili; therefore, the findings may not fully represent the broader landscape of short-video health information. Finally, although we quantified comment counts, we did not analyze the sentiment or informational quality of user comments, which may overlook the risk of misinformation propagation through audience interactions.

To address these limitations and build on the current findings, future research could expand to multi-platform and multi-language comparisons and incorporate longitudinal designs to track changes in health information quality over time. Moreover, we propose the following practical recommendations for enhancing video quality:

Platform level: Implement a quality-labeling system for health-related videos, encourage creators to cite information sources, and prioritize the recommendation of professionally certified content.
Creator level: Strengthen digital media literacy training for healthcare professionals, encouraging them to produce well-structured, clearly narrated, and visually supported science communication videos—with particular attention to the scientific rigor and comprehensiveness of diagnosis- and therapy-related content.
User level: Enhance user-oriented feedback and guidance by conducting needs assessments to identify the core information demands of patients and families (e.g., post-operative rehabilitation, pain management) and aligning content production accordingly. Simultaneously, through features such as video labels and dedicated science channels, users should be guided to prioritize authoritative content, thereby improving their ability to discern information quality.

We believe that through multi-stakeholder collaboration, the reliability and public health value of health information dissemination on platforms such as TikTok can be enhanced, making them effective tools for supporting the management of chronic conditions in aging populations.

## Conclusion

This study evaluated 100 knee osteoarthritis (KOA)-related videos on TikTok. The results showed that the mean scores for DISCERN (36.29), JAMA (1.24), and GQS (2.45) were all at low levels, indicating overall insufficient informational quality and reliability. Although some videos may possess certain educational value in form, their content quality is uneven and carries a risk of misinformation; viewers are therefore advised to critically evaluate the source of information. Significant differences in video quality were observed across different sources (e.g., scores from science communicators were significantly higher than those from medical staff), suggesting that healthcare professionals need to further improve content quality and informational rigor when using social media for health communication. Furthermore, although the proportion of higher-quality videos has increased in recent years, the overall quality distribution shows a trend toward polarization, with lower-quality content still predominating. It is recommended that platforms or relevant institutions strengthen content review and quality guidance. Given the broad influence of social media in disseminating health information, further research remains necessary to promote the creation and dissemination of high-quality health content.

## Data Availability

The original contributions presented in the study are included in the article/Supplementary Material, further inquiries can be directed to the corresponding author.
